# Effects of Parental Dietary Restriction on Offspring Fitness in *Drosophila melanogaster*

**DOI:** 10.3390/nu15051273

**Published:** 2023-03-03

**Authors:** Hye-Yeon Lee, Bora Lee, Eun-Ji Lee, Kyung-Jin Min

**Affiliations:** Department of Biological Sciences and Bioengineering, Inha University, Incheon 22212, Republic of Korea

**Keywords:** dietary restriction, trans-generation effect, longevity, *Drosophila melanogaster*, aging

## Abstract

Dietary restriction (DR) is a well-established strategy to increase lifespan and stress resistance in many eukaryotic species. In addition, individuals fed a restricted diet typically reduce or completely shut down reproduction compared to individuals fed a full diet. Although the parental environment can lead to changes epigenetically in offspring gene expression, little is known about the role of the parental (F_0_) diet on the fitness of their offspring (F_1_). This study investigated the lifespan, stress resistance, development, body weight, fecundity, and feeding rate in offspring from parental flies exposed to a full or restricted diet. The offspring flies of the parental DR showed increases in body weight, resistance to various stressors, and lifespan, but the development and fecundity were unaffected. Interestingly, parental DR reduced the feeding rate of their offspring. This study suggests that the effect of DR can extend beyond the exposed individual to their offspring, and it should be considered in both theoretical and empirical studies of senescence.

## 1. Introduction

Since McCay et al. reported that the restriction of food intake increases the maximum lifespan in rats [1], dietary restriction (DR), i.e., reduced nutrient intake without malnutrition, has been well described as an intervention to delay aging in a wide array of organisms from yeasts to primates and to prevent the onset of age- or diet-associated diseases in rodents and primates [2]. DR significantly impacts the life-history characteristics of organisms, including development, reproduction, locomotion, and lifespan. Altered life-history characteristics by the parental environment can lead to epigenetic modification in offspring gene expression. Life history theory asserts that a fundamental trade-off between the number of offspring tends to shift to “fewer but better-provisioned offspring” under stressful conditions [3]. In addition, the environmental programming of gene expression during gestation and early postnatal periods can produce long-term changes in the structure and function of an organism, allowing it to adapt better to its current environment [4,5]. Thus, DR may alter the phenotype of individuals exposed directly to DR and their offspring via parental effects [6,7].

Numerous studies have reported that the parental diet of rodents affects the fitness of their offspring. In most of these studies on rodents, however, a dietary regimen has been applied only during gestation and lactation [8,9,10,11]. Most studies on the effects of the parental diet during pre-pregnancy focused on the negative effects of obese parents [12,13,14] or the transgenerational effects of parental DR. For example, some researchers reported that a low maternal protein diet during pregnancy leads to reduced lifespan [8] or hyperinsulinemia and lowered insulin-signaling protein expression [9] in rat offspring or changes in the mitochondria gene expression in the liver and skeletal muscle of mice offspring [11]. Restricted diet during pregnancy also increased the low-density lipoprotein and global histone H3 acetylation but did not change the hepatic DNA methylation and expression in the offspring of rat fetuses [15,16,17]. Although the transgenerational effects of pre-pregnancy diets in vertebrates are still lacking, there are several studies of the transgenerational effects of DR in invertebrate model studies. In fruit flies, high sugar levels in the parental diet influence offspring obesity [12] or the obese-like phenotype [13], but parental sugar consumption does not affect the offspring’s lifespan [18]. In nematodes, the normal diet-fed offspring of a fasting-induced DR mother showed reduced fecundity, slower growth rate, decreased body size, and/or decreased mortality [19,20]. Although there is increasing evidence that the father’s diet or fitness also influences fitness in their offspring [18,21,22,23], most previous studies have focused only on the maternal DR effects and studies examining both maternal and paternal effects in a single experimental design are limited. In addition, the effect of early parental DR without fasting on offspring fitness is still unclear.

This study examined the effects of parental DR on the lifespan and fitness of their offspring using *Drosophila melanogaster*, one of the most widely used model organisms for studies on genetics and transgenerational effects. In this study, we investigated the intergenerational effect of DR (F_0_–F_1_) since previous studies suggested that the longevity effect of DR is instant, not persisting for multiple generations [20,24,25]. The results showed that short periods of parental DR increased the lifespan, resistance to environmental stress, and body weight but had little effect on the development, fecundity, and feeding rate of the offspring.

## 2. Materials and Methods

### 2.1. Fly Strain and Husbandry

The experiments were conducted using wild-type Canton-S flies, originally obtained from the Bloomington *Drosophila* Stock Center (Indiana University, Bloomington, IN, USA). All flies were cultured and reared at 25 °C and 65% humidity on 12:12 h light:dark cycles. Larval crowding was avoided by laying approximately 150 eggs on 250 cm^3^ fly bottles containing 25–30 mL of medium and were developed until the eclosion to adult. Standard CSY medium (52 g/L cornmeal, 110 g/L sugar, 25 g/L baker’s yeast, 8 g/L agar, 5 mL/L propionic acid, and 2.2 mL/L tegosept in 95% ethanol) was used to rear the fly larvae. Parental DR was administered by feeding the parent flies CSY food, including 160 g/L (full diet) or 40 g/L (restricted diet, 25% protein level of full diet) Saf-yeast extract.

### 2.2. Fly Collection and Parental Breeding Design

**Figure 1:**
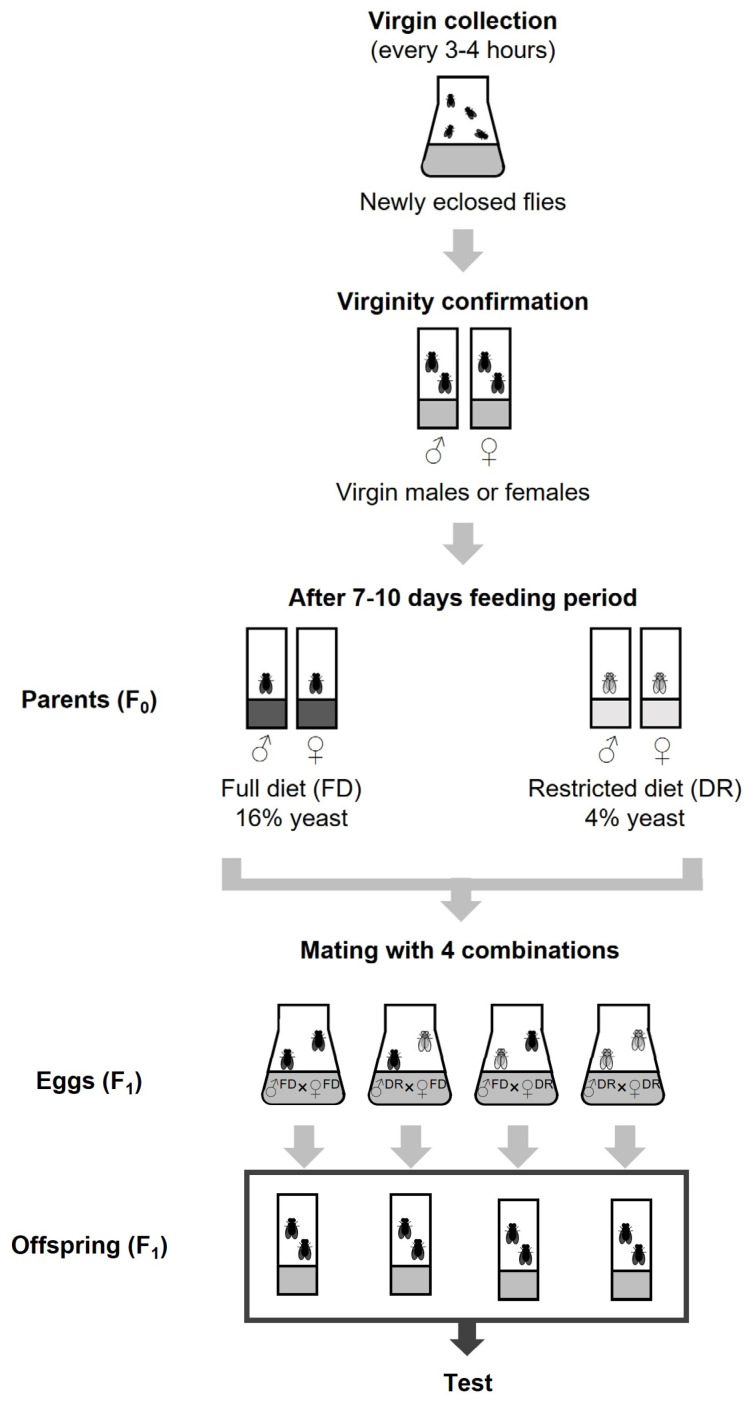
Schematic outline for methods to treat the medium for parents (F_0_) and offspring (F_1_). Newly eclosed flies for the parental generation were collected within 12 h and allocated to vials containing full diet (FD) or restricted diet (DR) medium. After a 7–10 day feeding period, parental flies were allocated to obtain eggs (F_1_) in the standard CSY medium. The number of parent flies used was 5 to 10 pairs in a full-fed diet group and 10 to 15 pairs in a restricted-fed diet.

Newly eclosed flies for parental generation were collected within 12 h and allocated to Φ25 × 100 mm vials containing full or restricted medium. Approximately 20 single-sex flies were kept per vial. The flies were transferred to fresh vials twice a week. After a 7–10 days feeding period, the parental flies were allocated to obtain eggs in standard CSY medium. The larval density of the offspring was adjusted by controlling the number of mating pairs of parents because the larval viability is influenced by larval density [26]. The number of parent flies used was 5 to 10 pairs in a full-fed diet group and 10 to 15 pairs in a restricted diet. Figure 1 outlines the experimental scheme.

### 2.3. Lifespan

Newly eclosed Canton-S adult flies were collected over 48 h and assigned randomly to a 500 cm^2^ demography cage to a final density of 100 males and 100 females per cage. All flies were cultured at 25 °C and 65% humidity on 12:12 h light:dark cycles. The fresh food was changed every two days, and all deaths were recorded. Three replicates were established for each group. Four trials were conducted to confirm the offspring’s lifespan. The parental flies were kept in mixed-sex groups similar to natural conditions (trials I and II) or in single-sex groups eliminating the reproductive effects (trials III and IV) to consider sexual interactions. The Kaplan–Meier survival estimator was used to estimate the survival function from the lifetime data. Log-rank tests were carried out to determine the statistical significance of the differences in the mean lifespan. In this study, maximum lifespan is defined as when the last 25% of the fruit flies used in the experiment are alive. The JMP statistical package (SAS Institute, Cary, NC, USA) and Statistical Package for the Social Sciences (SPSS, SPSS Inc., Chicago, IL, USA) were used for the analyses.

### 2.4. Stress Resistance

Seven- to ten-day-old offspring flies were transferred into a new vial (Φ25 × 100 mm) with ten single-sex flies in each. Fifteen vials were established for each group in each stress resistance test. All flies were tested at 25 °C and 65% humidity on 12:12 h light:dark cycles. For the stress resistance test with heat shock, the F_1_ flies were exposed to 39.5 °C by transferring heat through the air. The number of dead flies was recorded every 10 min until all the flies had died. For the starvation resistance test, the F_1_ flies were exposed to a medium that contained only 8 g/L agar. The number of dead flies was recorded every six hours until all the flies had died. The F_0_ flies were exposed to SY medium for the oxidative stress resistance test. The F_1_ flies were exposed to acute oxidative stress (18 mM paraquat). The food for oxidative stress contained 50 g/L sucrose (Sigma–Aldrich, St. Louis, MO, USA) to exclude the effects of starvation. The number of dead flies was recorded every three hours until all the flies had died. The Kaplan–Meier survival estimator was used to estimate the survival function from the lifetime data. Log-rank tests were carried out to determine the statistical significance of the differences in the mean lifespan. The JMP statistical package (SAS Institute, Cary, NC, USA) was used for the analyses.

### 2.5. Developmental Viability and Time

The 2-day-old flies were put in egg collection plate (90 × 15 mm diameter) containing standard CSY with 4% agar, and the eggs were collected for 12 h using the fast egg collection method. Then, 10 eggs were gently transferred to Φ25 × 100 mm vials containing standard CSY medium using a small brush. All flies were cultured at 25 °C and 65% humidity on 12:12 h light:dark cycles. The pupae and adult flies were counted every 12 h until no additional flies emerged. Fifteen replicates were established for each group. The data are presented as the mean ± SEM values.

### 2.6. Fecundity

Newly eclosed virgin flies were collected, and 24 h later, 1 female and 2 males were placed together in the Φ25 × 100 mm vial containing CSY medium. All flies were tested at 25 °C and 65% humidity on 12:12 h light:dark cycles. The flies were transferred carefully every 24 h, and the number of eggs laid on the medium was counted for 10 days. Twenty replicates for each group were established. The data are presented as the mean ± SEM.

### 2.7. Feeding Rate

The flies were pre-exposed to starvation for four hours before feeding. All flies were tested at 25 °C and 65% humidity. The 20-day-old-flies were fed a standard CSY medium containing 2.5% FD&C blue No.1 for 1 hour. The fly heads were cut off by quick freezing in liquid N_2_ to remove the red eyes of a fly. Then, 4 flies were placed in a 1.5 mL tube and homogenized with 100 µL of distilled water. The samples were centrifuged at 13,000 r/min (8400 × *g*) for 5 minutes at 4 °C. Subsequently, 100 µL of supernatant of the sample without lipid was transferred to another 1.5 mL tube and 100 µL distilled water was added. The absorbance was measured at 595 nm using a Sunrise microplate reader (TECAN, Männedorf, Switzerland). Eighteen replicates were established for each group. The data are presented as the mean ± SEM.

### 2.8. Body Weight

Newly eclosed flies were collected and transferred to Φ25 × 100 mm vials containing 10 single-sex flies each. All flies were reared at 25 °C and 65% humidity on 12:12 h light:dark cycles. The body weight of the flies was measured on a microbalance (PAG214C, Ohaus, Parsippany, NJ, USA) after CO_2_ anesthesia. After seven days on CSY foods, the body weight of the same flies was measured. Fifteen replicates were established for each group. The data are presented as the mean ± SEM.

### 2.9. Statistical Analysis

The log-rank tests for the lifespan and stress resistance data were carried out using survival models (Kaplan–Meier survival analysis) in the IBM SPSS statistics 21 (IBM, Armonk, NY, USA). The test for normality (Shapiro–Wilk test) and the statistical probabilities (F-test, t-test, and Wilcoxon rank sum test) of the data in this study were performed using R studio software.

## 3. Results

### 3.1. Dietary Restriction Increases the Lifespan and Reduces the Fecundity in Parent Flies

**Figure 2:**
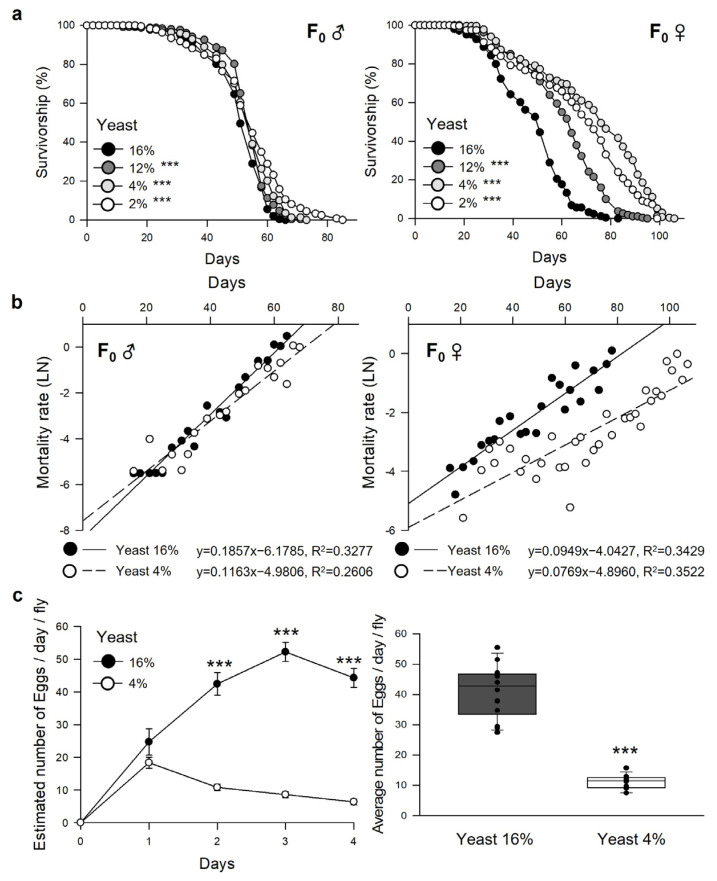
Effects of dietary restriction on lifespan and fecundity of parental (F_0_) flies. (**a**,**b**) Lifespan (**a**) or mortality rate (**b**) of F_0_ males (left, F_0_ ♂) and F_0_ females (right, F_0_ ♀) fed on a CSY medium with 16%, 12%, 4%, or 2% yeast extract. *** *p* < 0.0001; Log-rank test vs. 16%. (**c**) Number of egg production per day for 4 days (left), and the average number of eggs for 4 days (right) of F_0_ females fed on a full diet (16%) or restricted diet (4%). The error bars correspond to the standard error of the mean (SEM) among replicates (n = 20). The lifespan of F_0_ flies is presented with black (16%), dark gray (12%), gray (4%), or white (2%) circles. The mortality rate and egg production per day of F_0_ flies are presented with black (16%) or white (4%) circles or boxes. *** *p* < 0.0001; Student’s *t*-test (one-, two-day, and the average number of eggs) or Wilcoxon rank sum test (three- and four-day).

To determine optimal DR conditions in our laboratory conditions, parent fruit flies (F_0_) were fed a 2, 4, 12, or 16% yeast extract. The F_0_ male flies fed the 2 or 4% yeast extract diet had higher survival than those fed the 16% yeast diet (Figure 2a, Table 1, 16%, 50.89 ± 0.57 days; 12%, 53.64 ± 0.53 days, 5.4% increase, log-rank test, χ^2^ = 13.50, *p* < 0.0005; 4%, 53.21 ± 0.66 days, 4.6% increase, log-rank test, χ^2^ = 19.01, *p* < 0.0001; 2%, 53.05 ± 0.78 days, 4.2% increase, log-rank test, χ^2^ = 28.86, *p* < 0.0001). The F_0_ female flies fed the 2 or 4% yeast extract diet also had higher survival than those fed the 16% yeast diet (Figure 2b, Table 1, 16%, 47.66 ± 0.91 days; 12%, 60.41 ± 1.01 days, 26.8% increase, log-rank test, χ^2^ = 105.47, *p* < 0.0001; 4%, 72.36 ± 1.39 days, 51.8% increase, log-rank test, χ^2^ = 225.84, *p* < 0.0001; 2%, 66.73 ± 1.36 days, 40.0% increase, log-rank test, χ^2^ = 175.00, *p* < 0.0001). In the F_0_ females, however, the lifespan of the flies fed 2% yeast was 7.78% shorter than the flies with 4% yeast, indicating that this 2% yeast diet could lead to malnutrition in fruit flies [27,28]. Thus, 16% yeast was considered the full diet (FD), and 4% was the restricted diet (DR) for parental diet conditions.

DR in individuals reduced reproduction. The fecundity of F_0_ females was measured and adjusted for the number of eggs between the F_0_ groups fed an FD or DR to remove the possibility that reduced offspring population affects the physiological changes. As described elsewhere, DR reduced the fecundity of F_0_ females (Figure 2c; full diet, average number of eggs/days = 41 ± 2.3; restricted diet, average number of eggs/days = 11 ± 0.6, 73.1% decrease, student’s *t*-test, *p* < 0.0001). According to these results, the number of parent flies was controlled to 5 to 10 pairs in the full-fed diet group and 10 to 15 pairs in DR to obtain the offspring fruit flies (F_1_). Figure 1 shows the detailed mating scheme.

### 3.2. Parental Dietary Restriction Increases the Lifespan of the Offspring Flies

**Figure 3:**
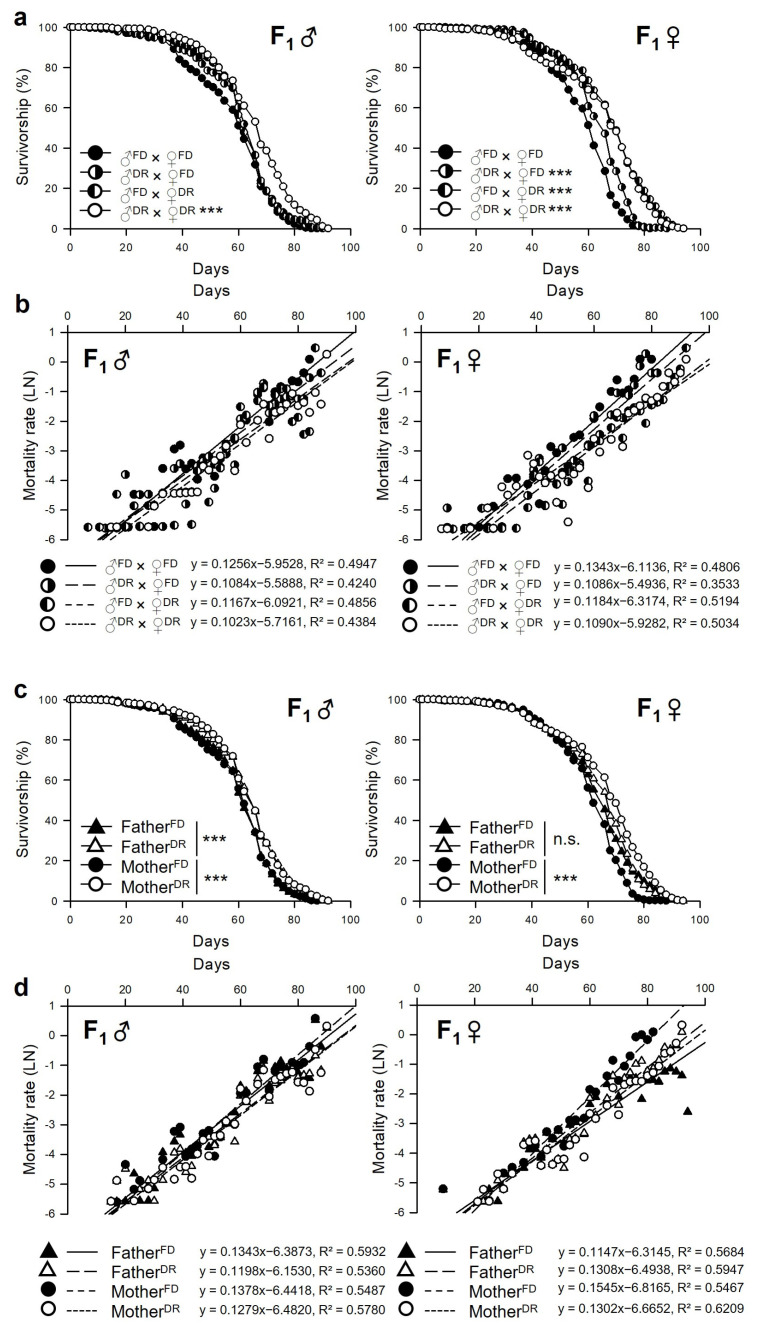
Effects of parental (F_0_) DR on offspring (F_1_) lifespan in *D. melanogaster*. (**a**,**b**) Lifespan (**a**) and mortality rate (**b**) of F_1_ male (left, F_1_ ♂) and female (right, F_1_ ♀) from parents exposed to a full diet (FD) or restricted diet (DR) with four combinations of flies: FD male and FD female (♂^FD^ × ♀^FD^), DR male and FD female (♂^DR^ × ♀^FD^), FD male and DR female (♂^FD^ × ♀^DR^), and DR male and DR female (♂^DR^ × ♀^DR^). Lifespan and mortality rate of F_1_ flies is presented with black (♂^FD^ × ♀^FD^), circles split in half (♂^DR^ × ♀^FD^ or ♂^FD^ × ♀^DR^), or white (♂^DR^ × ♀^DR^) circles. *** *p* < 0.0001; Log-rank test vs. ♂^FD^ × ♀^FD^. (**c**,**d**) Lifespan (**c**) and mortality rate (d) of F_1_ males (left, F_1_ ♂) and females (right, F_1_ ♀) from parents exposed to a full diet (FD) or restricted diet (DR). The triangles indicate the data of F_1_ flies analyzed based on the father’s diet, and the circles indicate the data of F_1_ flies analyzed based on the mother’s diet. The black indicates the data of F_1_ flies from parents with FD, and the white indicates the data of F_1_ flies from parents with DR. *** *p* < 0.0001; Log-rank test vs. full diet parent (Father^FD^ or Mother^FD^).

Four combinations of flies were used to determine if the longevity effect of parental DR affects the lifespan of their F_1_ flies: FD male and FD female (♂^FD^ × ♀^FD^), DR male and FD female (♂^DR^ × ♀^FD^), FD male and DR female (♂^FD^ × ♀^DR^), and DR male and DR female (♂^DR^ × ♀^DR^) were mated (Figure 1). The lifespan of F_1_ flies was measured under normal diet conditions (Figure 3). The F_1_ lifespan was increased, and the F_1_ mortality was decreased when their parents were fed on a DR instead of FD (Figure 3a,b and Table 2; Male, ♂^FD^ × ♀^FD^, 55.87 ± 0.89 days; ♂^DR^ × ♀^DR^, 63.42 ± 0.88 days, 13.5% increase, log-rank test, χ^2^ = 43.3299, *p* < 0.0001; Female, ♂^FD^ × ♀^FD^, 56.04 ± 0.78 days; ♂^DR^ × ♀^DR^, 63.20 ± 1.03 days, 13.3% increase, log-rank test, χ^2^ = 92.7941, *p* < 0.0001). Interestingly, cross-combination with the FD and DR (♂^DR^ × ♀^FD^ or ♂^FD^ × ♀^DR^) extended the lifespan of only the F_1_ female (♂^DR^ × ♀^FD^, 60.50 ± 0.74 days, 8.0% increase, log-rank test, χ^2^ = 24.138, *p* < 0.0001; ♂^FD^ × ♀^DR^, 65.49 ± 0.92 days, 16.9% increase, log-rank test, χ^2^ = 109.4231, *p* < 0.0001) but not the F_1_ male (♂^DR^ × ♀^FD^, 60.50 ± 0.74 days, log-rank test, χ^2^ = 3.6747, *p* = 0.0552; ♂^FD^ × ♀^DR^, 58.80 ± 0.86 days, log-rank test, χ^2^ = 3.6341, *p* = 0.0566). The results were re-analyzed with the paternal or maternal diet to clarify which one of the father or mother affects the F_1_ lifespan. Father^FD^ included the ♂^FD^ × ♀^FD^ and ♂^FD^ × ♀^DR^, Father^DR^ included the ♂^DR^ × ♀^FD^ and ♂^DR^ × ♀^DR^, Mother^FD^ included the ♂^FD^ × ♀^FD^ and ♂^FD^ × ♀^FD^, and Mother^DR^ included the ♂^FD^ × ♀^DR^ and ♂^DR^ × ♀^DR^ (Figure 3c,d and Table 3). The lifespan of an F_1_ male that had a DR-fed father or mother increased regardless of what the opposite parent was fed (Father^FD^, 57.33 ± 0.62 days; Father^DR^, 60.78 ± 0.63 days, 6.0% increase, log-rank test, χ^2^ = 24.2517, *p* < 0.0001; Mother^FD^, 57.06 ± 0.63 days; and Mother^DR^, 61.10 ± 0.62 days, 7.1% increase, log-rank test, χ^2^ = 25.3409, *p* < 0.0001). In females, only the DR-fed mother increased the lifespan; the father’s diet did not affect the lifespan of the F_1_ female (Father^FD^, 60.90 ± 0.64 days; Father^DR^, 61.85 ± 0.64 days, 1.6% increase, log-rank test, χ^2^ = 0.7762, *p* = 0.3783; Mother^FD^, 58.31 ± 0.55 days; and Mother^DR^, 64.35 ± 0.69 days, 10.4% increase, log-rank test, χ^2^ = 137.0076, *p* < 0.0001). Thus, in the case of the female offspring, the longevity effect by parental DR was more prominent than that of the male offspring, but it appears to be influenced mainly by the mother’s diet. On the other hand, the male offspring tended to extend their lifespan even if only one of the parents ate the restricted diet, but it showed a significant increase in lifespan when both parents restricted the diet.

The lifespan of F_1_ was measured independently in three repeated trials (Figure S1). These parental DR-induced longevity effects were also shown when the yeast type of the parental diet was changed from yeast extract to Brewer’s inactive yeast (Figure S1a). The mean lifespan of flies from parents fed on a DR tended to increase in the F_1_ males and females in all trials. In particular, the F_1_ females from parents fed a DR had significantly longer lifespans in all trials. Parents fed a DR tended to increase the lifespan of the F_1_ males, but only two trials (first and third) were statistically significant. Overall, seven days of exposure to different dietary regimens in parental flies had significant effects on the F_1_ lifespan, and there were also sex-specific effects on the offspring’s lifespan. Accordingly, subsequent experiments were performed using only the FD × FD and DR × DR groups to investigate the effects of parental DR on offspring by removing maternal–paternal factors.

### 3.3. Parental (F_0_) Dietary Restriction Increases the Resistance to Various Stressors in Offspring (F_1_) Flies

**Figure 4:**
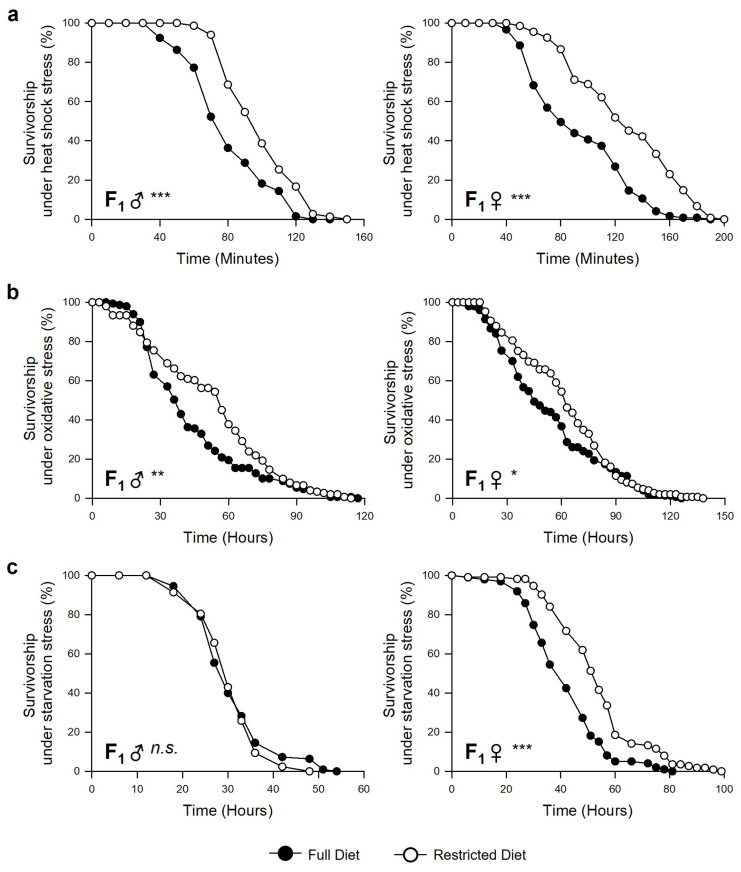
Effects of parental (F_0_) DR on the resistance to environmental stressors in offspring (F_1_) male (left, F_1_ ♂) and female (right, F_1_ ♀). (**a**) Heat shock stress resistance of F_1_ male (left, F_1_ ♂) and female (right, F_1_ ♀). (**b**) Oxidative stress resistance of F_1_ male (left, F_1_ ♂) and female (right, F_1_ ♀). (**c**) Starvation resistance of F_1_ male (left, F_1_ ♂) and female (right, F_1_ ♀). The black circles indicate the lifespan of F_1_ flies from parents with FD, and the white circles indicate the lifespan of F_1_ flies from parents with DR. * *p* < 0.05; ** *p* < 0.005; *** *p* < 0.0001; Log-rank test (n = 150).

The effects of parental diet on the F_1_ susceptibility to environmental stresses were examined because resistance to environmental stress is closely related to individual longevity. The survival rate of the offspring under heat shock stress, oxidative stress, or starvation stress was evaluated. The resistance to heat shock was significantly greater in both male and female offspring from the parents fed on a DR (24% and 37%, respectively) than the offspring from parents fed on an FD (Figure 4a, Log-rank test, male, χ^2^ = 39.0, *p* < 0.0001; female, χ^2^ = 50.0, *p* < 0.0001). The resistance to oxidative stress was also increased by 18% and 17% in the male and female offspring of parents fed a DR, respectively, than offspring from parents fed on an FD (Figure 4b, Log-rank test, male, χ^2^ = 6.7, *p* < 0.01; female, χ^2^ = 4.1, *p* < 0.05). In the case of starvation stress, parental DR increased resistance to starvation in female offspring by 23% but not in male offspring (Figure 4c, Log-rank test, male, χ^2^ = 1.0, *p* = 0.316; female, χ^2^ = 33.6.0, *p* < 0.0001). Thus, stress resistance was generally greater in F_1_ from parents fed a DR than those from parents fed on an FD. Moreover, there also appeared to be sex-specific effects on offspring similar to the results of the lifespan tests. These results suggest that parental DR has beneficial effects on various environmental stress resistances of their offspring.

### 3.4. Parental Dietary Restriction Affects the Offspring’s Body Weight and Feeding Rate but Not the Development and Fecundity of the Offspring Flies

**Figure 5:**
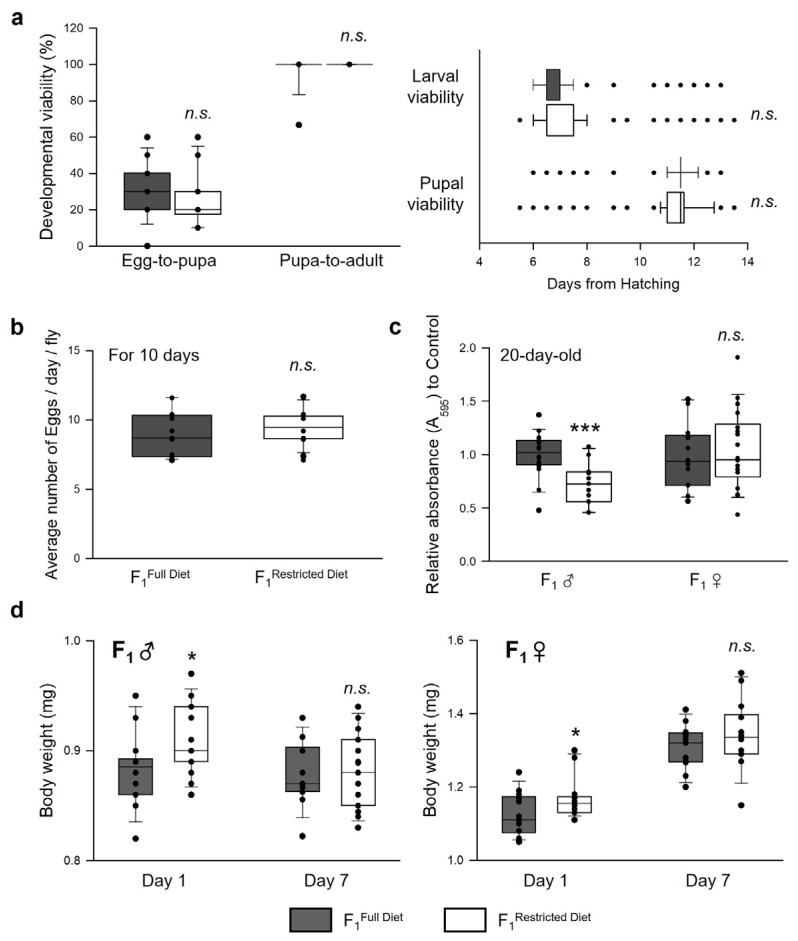
Effects of parental (F_0_) DR on development, fecundity, feeding behavior, and body weight of the offspring (F_1_) flies. (**a**) Developmental viability from egg to pupa, or from pupa to adult (left) and developmental timing to emergence (right) of F_1_ flies (n = 150). (**b**,**c**) Fecundity (**b**) and feeding rate (c) of F_1_ flies. The error bars correspond to the standard error of the mean (SEM) among replicates. *** *p* < 0.0001; Student’s *t*-test (Fecundity n = 20; feeding rate n = 18). (**d**) Adult body weight of one- or seven-day-old F_1_ male (left, F_1_ ♂) and female (right, F_1_ ♀). The error bars correspond to the standard error of the mean (SEM) among replicates. * *p* < 0.05; Student’s *t*-test (one-, seven-day-old F_1_ male, and seven-day-old F_1_ female; n = 150) or Wilcoxon rank sum test (one-day-old F_1_ female; n = 150). The black boxes indicate the lifespan of F_1_ flies from parents with FD, and the white boxes indicate the lifespan of F_1_ flies from parents with DR.

The offspring’s developmental viability, body weight, fecundity, and feeding rate, which can influence the ability to survive and thrive in the environment, were measured to examine the intergenerational effects of parental DR on various offspring (F_1_) traits. DR leads to delayed developmental timing and reduced fecundity, but the parental diet did not affect their offspring’s developmental viability (Figure 5a left, Wilcoxon rank sum test; egg-to-pupa, *p* = 0.124; pupa-to-adult, *p* = 0.164), developmental timing (Figure 5a right, Wilcoxon rank sum test; emergence to pupa, *p* = 0.361; emergence to adult, *p* = 0.401), and fecundity (Figure 5b, Student’s *t*-test, *p* = 0.382). The feeding rate of their offspring was measured to determine if parental feeding behaviors enhanced by DR were transmitted to the offspring. Interestingly, parental DR decreased the feeding rate of F_1_ males but not F_1_ females (Figure 5c; F_1_ ♂, Student’s *t*-test, *p* < 0.0001; F_1_ ♀, Student’s *t*-test, *p* = 0.733). The offspring’s body weight was then measured because it has been reported that parental diet could alter the body composition of their offspring [13]. In these results, newly hatched young adult male or female offspring (1 day of age) were heavier when their parents were fed DR than when their parents were fed the FD (Figure 5d; F_1_ ♂, Day 1, Student’s *t*-test, *p* < 0.05; F_1_ ♀, Day 1, Wilcoxon rank sum test, *p* < 0.05). These differences in the body weight at the 1-day-old age disappeared at the 7-day-old age (Figure 5d; F_1_ ♂, Day 7, Student’s *t*-test, *p* = 0.366; F_1_ ♀, Day 7, Student’s *t*-test, *p* = 0.289). Interestingly, the offspring of the 2 groups showed a difference in the rate of body weight change over 7 days in the same medium composition (male, F_1_^Full Diet^, 0.37% decrease, F_1_^Restricted Diet^, 9.37% decrease; female, F_1_^Full Diet^, 16.68% increase, F_1_^Restricted Diet^, 14.92% increase). It indicates that the offspring of DR-fed parents have more difficulty gaining weight than the offspring of FD-fed parents. Thus, the offspring’s body weight was increased by parental DR, but there were no changes in developmental time. Hence, the effects of reduced food intake might be a factor that leads to a longevity effect by parental DR because lifespan extension can be induced by the reduction of food intake [29].

## 4. Discussion

This study examined the effects of parental (F_0_) dietary restriction on the offspring (F_1_) fitness, such as lifespan, resistance to environmental stress, development, body weight, fecundity, and feeding rate, using the fruit fly, *Drosophila melanogaster*. In this study, we investigated intergenerational (F_0_–F_1_) effects of DR because previous studies indicated DR effects did not persist for several generations. Great-grand-offspring (F_3_) of fasting-induced DR mother showed a reduced fitness and increased mortality risk indicating that the transgenerational benefit of DR is instant in nematode [20]. Similarly, after 25 generations of DR, male DR flies had increased reproduction, but their survival rate did not increase [24]. In the case of females, after 50 generations of DR, female DR flies had increased reproduction, but their survival rate decreased [25]. These results indicate that longevity effects induced by DR may not persist over multiple generations, and organisms evolve to increase reproductivity under long-term DR conditions. This study showed that short periods of parental dietary restriction during the adult stage had a significant impact on fitness changes, particularly the lifespan in offspring flies (Figure 3). The offspring phenotype could be altered in response to adaptive parental effects, which might explain why lifespan extension occurred by reducing the intrinsic mortality in the individuals and their offspring. The lifespan and environmental stress resistance are correlated [30,31]. The offspring’s resistance to heat shock, starvation, or oxidative stress was examined in this study (Figure 4). Both male and female offspring from parents fed a restricted diet survived better than offspring from parents fed a full diet, except for survival with starvation resistance in male offspring. The intergenerational DR effects on lifespan and resistance to starvation stress were sex specific. Previous studies reported that parental environmental conditions might induce different epigenetic effects in sons versus daughters. For example, exposure to environmental compounds, such as endocrine disruptor vinclozolin, induced sex-specific transgenerational alterations in the brain transcriptomes and behavior in rats [32]. In a study using the springtail *Orchesella cincta*, similar to our results, the offspring of a DR-fed mother showed increased flexibility in low-food conditions, but these maternal effects were not observed in the sons [33]. Heat shock proteins (HSPs) are induced by several stressors, such as heat shock, oxidative stress, or even DR [34]. These proteins perform a chaperone function by assisting new proteins in correcting folding or refolding proteins damaged by cell stress [35]. DR, as hormetic metabolic stress, can activate several defense mechanisms to increase the lifespan and could induce the heat shock response [36]. For example, DR promoted the HSP70 protein and *hsp*70 mRNA synthesis in hepatocytes of 28-month-old rats [34]. Offspring from parents in the Artemia model who survived heat shock have greater tolerance to thermal stress than their respective offspring controls [37]. Thus, the parental DR can lead to compensatory effects related to extended lifespan and increased environmental stress resistance in the offspring. The theory of hormesis explains that a stressful circumstance could enhance the organism’s fitness after exposure [38].

The thrifty phenotype hypothesis is conceptually associated with hormesis, and these epigenetic effects can be passed from one generation to the next [39]. This hypothesis explains how flies might quickly and semi-permanently modify gene expression to adapt to a restricted regimen and pass these effects on to the next generation, thereby increasing the offspring’s survival by increasing their stress resistance. Several studies showed prenatal DR significantly changed the expression of major genes related with two epigenetic mechanisms (DNA methylation and histone modification) [15,16,17]. We investigated the expression of the *sir2* gene, a well-known longevity effecter of DR, but the *sir2* gene expression did not increase in the offspring of DR-fed parents (Figure S2). Previous studies reported that parental diet affects offspring’s energy metabolism, fat content, glucose homeostasis, and insulin resistance [40,41,42]. Thus, future studies are necessary to investigate the epigenetic effects of DR on energy metabolism, including fat and glucose homeostasis, to improve our understanding of the mechanisms of beneficial DR effects.

Increased resistance to starvation might be connected to the increased body weight of the offspring since body weight and starvation resistance are tightly related to each other. Our results showed that parents fed a restricted diet produced heavier offspring than those fed a full diet at birth (Figure 5d). Similarly, the offspring from parents fed a restricted diet showed increased body size [43] or body mass [44] in fruit flies. Given the well-known intraspecific trade-off between the number of offspring and offspring size [3,45], parents fed a restricted diet produce larger offspring. This outcome might be one of the mechanisms of adaptive parental effects [46]. In organisms without parental care, parental provisioning can be estimated by their offspring’s egg or newborn size [47]. However, the differences in body weight observed between the two groups of newly eclosed offspring disappeared after seven days in both sons and daughters (Figure 5d), suggesting that the effect of the parental diet on the offspring’s body weight is not sustained to the early adult stage of the offspring. In a mice study, interestingly, there was a similar result that food restriction of obese mice during pregnancy induced decreased body weight gain of offspring compared to that of high-fat diet mother’s offspring [48]. Additionally, many rodent studies showed that restricted protein content is associated with higher food intake in female offspring, but these differences in food intake disappeared when food intake was adjusted for body mass [49]. These results indicate that parental DR can reduce the risk of obesity in offspring as well as parents.

According to this present study, developmental viability from egg-to-pupa (pupation) or pupa-to-adult (eclosion) and developmental time to emergence showed no differences between the groups by their parental diet. Nevertheless, previous studies reported several contradictive effects of parental diet on offspring development in fruit fly or nematodes. The eggs from parents raised on a poor larval diet (approximately 1/4 to 1/8 of the standard diet) were heavier and developed faster than those from parents raised on standard food [44,46,50], but there are strain-specific differences in development time [44]. In contrast, the offspring from both parents raised on a poor diet have a longer development time than those from parents raised on standard food [43,44]. Interestingly, developmental time of the offspring was shortened when only one parent was raised on a poor diet [28] indicating that both maternal and paternal nutrition can influence offspring development. In the nematode, female offspring of fasting-induced DR mother showed reduced fecundity, slowed development time, and decreased body size [19,20]. On the other hand, most previous studies used “poor and standard” levels of dietary regimens or fasting-induced DR, but this study used full-fed and dietary restriction (reduced nutrient intake, especially yeast-protein source, without malnutrition) conditions. Additionally, this study adjusted the number of eggs between the F_0_ groups fed an FD or DR to remove the possibility that offspring population affects the physiological changes. Thus, the differences in the results of the previous and present studies might be due to the difference in fly strain, species, dietary composition, and/or population density control.

Parental DR reduced the food intake of their male offspring, but there were no significant changes in that of the female offspring (Figure 5c). This difference might be due to sexual dimorphic effects on feeding behavior. Because the lifespan is influenced significantly by food intake [29], reduced food consumption in male offspring whose parents were exposed to a restricted diet might be one factor that leads to an extended lifespan. Several reports suggested that transgenerational feeding behavior of offspring is influenced by parental dietary regimen in rat or fruit fly [51,52,53]. Several studies using fruit flies have shown that the longevity effect of DR is more effective in females than in males [54,55]. This may be because male and female generally pursue divergent reproductive strategies and thus have different sensitivities to nutritional interventions [56,57]. Therefore, the intrinsic changes in the mothers that led to the greater lifespan extension by DR may have been passed on to their daughters also. Our results suggest that the feeding behavior may be a heritable predisposition to their offspring, and the effects of reduced food intake might be one of the factors that lead to their extended lifespan.

## 5. Conclusions

Previous studies revealed the effects of diet restriction on aging, with lifespan extension and other beneficial effects observed in flies, nematodes, yeasts, insects, rodents, and possibly even primates [2]. The underlying mechanisms of hormetic DR effects on cellular responses remain conserved across the evolutionary tree, even though many organisms have diverged evolutionarily over time [58]. At the same time, theoretical models show that maternal effects can influence the speed and trajectory of evolutionary changes [59]. In this light, it would be worth determining if the parental effects of DR observed here apply to other organisms and developing an explicit theoretical treatment of the role of parental effects on the evolution of senescence.

## Figures and Tables

**Table 1:** Lifespan and mortality of parents (F_0_) fed a 16%, 12%, 4%, or 2% yeast diet.

Sex	Yeast (%)	n	Mean-Lifespan			Change	Median-Lifespan	Change	Max. Lifespan (75% Failures)	Change	χ^2^	*p*-Value
Male	16	252	50.89	±	0.57		51		58			
	12	258	53.64	±	0.53	5.4%	55	7.8%	58	0.0%	13.5037	0.0002
	4	228	53.21	±	0.66	4.6%	55	7.8%	60	3.4%	19.0097	<0.0001
	2	238	53.05	±	0.78	4.2%	55	7.8%	62	6.9%	28.8639	<0.0001
Female	16	249	47.66	±	0.91		51		58			
	12	269	60.41	±	1.01	26.8%	64	25.5%	71	22.4%	105.4658	<0.0001
	4	269	72.36	±	1.39	51.8%	76	49.0%	91	56.9%	225.8365	<0.0001
	2	275	66.73	±	1.36	40.0%	71	39.2%	83	43.1%	175.0049	<0.0001

**Table 2:** Lifespan and mortality of male and female offspring (F_1_) from parents (F_0_) fed a full or restricted diet with four combinations.

Sex	Group	n	Mean-Lifespan			Change	Median-Lifespan	Change	Max. Lifespan (75% Failures)	Change	χ^2^	*p*-Value
Male	♂^FD^ × ♀^FD^	268	55.87	±	0.89		60		66			
	♂^DR^ × ♀^FD^	274	58.23	±	0.87	4.2%	62	3.3%	66	0.0%	3.6747	0.0552
	♂^FD^ × ♀^DR^	266	58.80	±	0.86	5.3%	60	0.0%	66	0.0%	3.6341	0.0566
	♂^DR^ × ♀^DR^	264	63.42	±	0.88	13.5%	66	10.0%	74	12.1%	43.3299	<0.0001
Female	♂^FD^ × ♀^FD^	267	56.04	±	0.78		60		66			
	♂^DR^ × ♀^FD^	278	60.50	±	0.74	8.0%	62	3.3%	70	6.1%	24.138	<0.0001
	♂^FD^ × ♀^DR^	283	65.49	±	0.92	16.9%	68	13.3%	76	15.2%	109.4231	<0.0001
	♂^DR^ × ♀^DR^	280	63.20	±	1.03	12.8%	68	13.3%	76	15.2%	92.7941	<0.0001

FD, Full diet; DR, restricted diet; FD male and FD female (♂^FD^ × ♀^FD^), DR male and FD female (♂^DR^ × ♀^FD^), FD male and DR female (♂^FD^ × ♀^DR^), and DR male and DR female (♂^DR^ × ♀^DR^).

**Table 3:** Lifespan and mortality of male and female offspring (F_1_) from parents (F_0_) fed a full or restricted diet.

Sex	Group	n	Mean-Lifespan			Change	Median-Lifespan	Change	Max. Lifespan (75% Failures)	Change	χ^2^	*p*-Value
Male	Father^FD^	534	57.33	±	0.62		60		66			
	Father^DR^	538	60.78	±	0.63	6.0%	62	3.3%	70	6.1%	24.2517	<0.0001
	Mother^FD^	542	57.06	±	0.62		60		66			
	Mother^DR^	530	61.10	±	0.62	7.1%	62	3.3%	70	6.1%	25.3409	<0.0001
Female	Father^FD^	550	60.90	±	0.64		62		70			
	Father^DR^	558	61.85	±	0.64	1.6%	66	6.5%	72	2.9%	0.7762	0.3783
	Mother^FD^	545	58.31	±	0.55		60		66			
	Mother^DR^	563	64.35	±	0.69	10.4%	68	13.3%	76	15.2%	137.008	<0.0001

FD, Full diet; DR, restricted diet.

## Data Availability

The datasets during and/or analyzed during the current study are available from the corresponding author upon reasonable request.

## Supplementary Materials

The following supporting information can be downloaded at: https://www.mdpi.com/article/10.3390/nu15051273/s1, Figure S1: Effects of parental (F_0_) DR on offspring (F_1_) lifespan of *D. melanogaster* in 2nd or 3rd trial; Figure S2: The mRNA levels of *sir2* genes in the male or female offspring.
