# Supplementary material for: Effects of Parental Dietary Restriction on Offspring Fitness in Drosophila melanogaster
Source: Nutrients. 2023 Mar 3;15(5):1273. doi: 10.3390/nu15051273 (PMC10005678; doi:10.3390/nu15051273)
Supplement: Supplementary file 1 [file nutrients-15-01273-s001.zip › nutrients-2176545-supplementary.pdf]

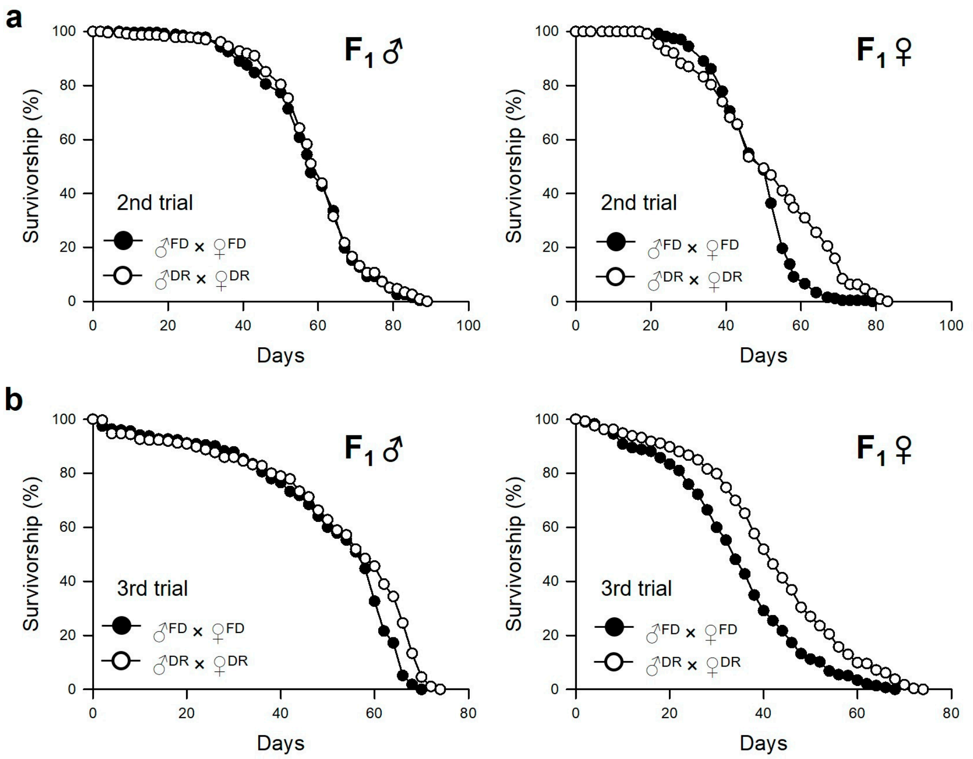

c

Trial	Sex	Group	n	Mean-lifespan	Change	Median-lifespan	Change	Maximum-lifespan	Change	χ^2	p-value
2nd	Male	♂FD × ♀FD	283	58.56 ± 0.79		58		67			
		♂DR × ♀DR	235	59.56 ± 0.87	1.7%	61	5.2%	67	0.0%	0.4602	0.4975
	Female	♂FD × ♀FD	275	48.34 ± 0.61		50		55			
		♂DR × ♀DR	239	51.50 ± 1.05	6.5%	50	0.0%	67	21.8%	31.3343	< 0.0001*
3rd	Male	♂FD × ♀FD	273	50.37 ± 1.03		58		62			
		♂DR × ♀DR	285	52.26 ± 1.11	3.7%	58	0.0%	66	6.5%	24.3173	< 0.0001*
	Female	♂FD × ♀FD	295	34.08 ± 0.82		34		44			
		♂DR × ♀DR	293	41.56 ± 0.91	21.9%	42	23.5%	52	18.2%	43.4017	< 0.0001*

Figure S1. Effects of parental (F_0) DR on offspring (F_1) lifespan of *D. melanogaster* in 2nd or 3rd trial.

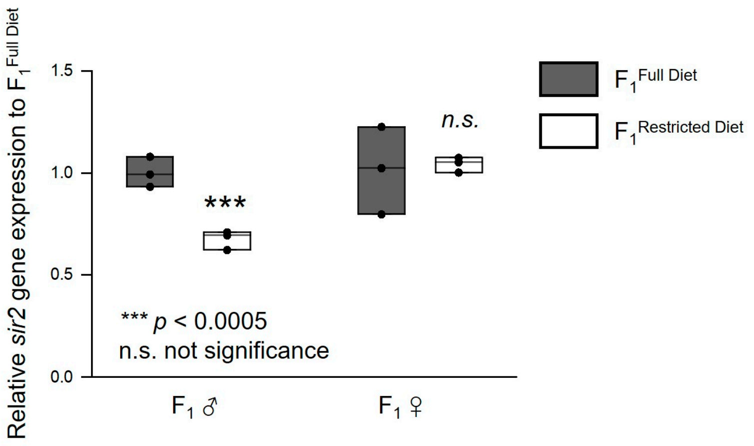

Figure S2. The mRNA levels of *sir2* genes in the male or female offspring.
